# Chitosan-Based Biomimetically Mineralized Composite Materials in Human Hard Tissue Repair

**DOI:** 10.3390/molecules25204785

**Published:** 2020-10-19

**Authors:** Die Hu, Qian Ren, Zhongcheng Li, Linglin Zhang

**Affiliations:** 1 State Key Laboratory of Oral Diseases & National Clinical Research Centre for Oral Disease, Sichuan University, Chengdu 610000, China; hudiehd@163.com (D.H.); 18380351309@163.com (Q.R.); lzcscu@163.com (Z.L.); 2 Department of Cariology and Endodontics, West China Hospital of Stomatology, Sichuan University, Chengdu 610000, China

**Keywords:** chitosan, biomimetic mineralization, template, composite materials, bone tissue engineering, dental hard tissue, repair

## Abstract

Chitosan is a natural, biodegradable cationic polysaccharide, which has a similar chemical structure and similar biological behaviors to the components of the extracellular matrix in the biomineralization process of teeth or bone. Its excellent biocompatibility, biodegradability, and polyelectrolyte action make it a suitable organic template, which, combined with biomimetic mineralization technology, can be used to develop organic-inorganic composite materials for hard tissue repair. In recent years, various chitosan-based biomimetic organic-inorganic composite materials have been applied in the field of bone tissue engineering and enamel or dentin biomimetic repair in different forms (hydrogels, fibers, porous scaffolds, microspheres, etc.), and the inorganic components of the composites are usually biogenic minerals, such as hydroxyapatite, other calcium phosphate phases, or silica. These composites have good mechanical properties, biocompatibility, bioactivity, osteogenic potential, and other biological properties and are thus considered as promising novel materials for repairing the defects of hard tissue. This review is mainly focused on the properties and preparations of biomimetically mineralized composite materials using chitosan as an organic template, and the current application of various chitosan-based biomimetically mineralized composite materials in bone tissue engineering and dental hard tissue repair is summarized.

## 1. Introduction

Bone and teeth are the two main hard tissues in the human body. Bone is a mineralized inorganic-organic composite, which is mainly composed of carbonated hydroxyapatite (HAP) and type I collagen [1]. Dentin, cementum, and enamel are hard structures of teeth. Dentin and cementum are both collagenous composites similar to bone, with apatite as the mineral phase [2,3]. However, enamel is quite special, as it is acellular, non-collagenous, and composed of 95–97% mineral by weight, with less than 1% organic material [4]. While there are differences in the composition and structure of these hard tissues, they are all organic-inorganic composites formed through biomineralization processes regulated by a series of cells and organic matrices (proteins, polysaccharides, etc.) [5,6].

Bone tissue engineering is now a popular and promising method for repairing bone defects due to the large-scale destruction or loss of bone tissue caused by events, such as trauma, infection, and tumor [7]. It is a technique involving the provision of three-dimensional scaffolds that act as artificial extracellular matrices, allowing cells to proliferate and maintain their specific functions, and serve as a template for new bone formations [8]. Numerous biomimetic scaffolds of different biomaterials have been applied in bone tissue engineering [9,10]. Similarly, when dental caries, trauma, or erosion cause defects of dental hard tissue, current clinical treatments cannot restore the original structure and properties of teeth. Some biomimetic materials and strategies that have appeared in recent years may be promising ways to fabricate enamel-like or dentin-like structures [11,12,13].

Chitosan, a natural cationic polysaccharide, has a similar chemical structure and biological behaviors to the components of the extracellular matrix (ECM) of bone and teeth. Chitosan has many biological properties, such as biocompatibility, biodegradability, polyelectrolyte action, etc. [14], which make it a suitable organic scaffold or template for the fabrication of organic-inorganic composites. Unlike simply mixing chitosan and inorganic minerals to construct composite materials, biomimetic mineralization technology, which is inspired by the biomineralization process, can deposit minerals in situ on chitosan organic templates, thereby constructing composite materials with closer structures and functions to those of bone or teeth. In recent years, chitosan-based composite materials fabricated by the biomimetic mineralization technique have been widely used in the field of bone tissue engineering and enamel or dentin biomimetic repair. Comparing with the artificial materials currently used to repair human hard tissues in clinic, such as ceramics, alloys, etc., chitosan-based materials have reduced costs and improved biocompatibility, with low possibility of causing allergic and inflammatory reactions in human body [15]. Besides, the bioactivities and mechanical properties of chitosan-based materials can also be improved with the addition of inorganic minerals [16]. 

In this article, we first briefly introduce the basic structure and properties of chitosan and then focus on the properties and preparation methods of chitosan-based biomimetically mineralized composite materials and their applications in human hard tissue repair in recent years. Since the composite materials are mainly composed of two parts—a chitosan organic template and inorganic minerals—we reviewed two aspects of the preparation methods of the composite materials: the preparations of various chitosan organic templates in composite materials and the biomimetic mineralization methods for preparing different inorganic minerals. Many aspects of the recent applications of chitosan-based biomimetically mineralized composite materials in the fields of bone tissue engineering and dental hard tissue repair were reviewed and presented in detail, including types of chitosan and minerals, composite forms, other organic/inorganic components, preparation techniques of chitosan templates, methods of biomimetic mineralization, and important properties of composite materials. 

## 2. Structure, Properties and Applications of Chitosan

Chitosan is a natural cationic polysaccharide that is obtained by the N-deacetylation of chitin, which is the second most ubiquitous polymer-after cellulose-on earth [17]. It is a linear copolymer composed of D-glucosamine (GlcN) and N-acetyl-d-glucosamine (GlcNAc), which are linked by the β-1, 4-glycosidic bond, with molecular weight ranges from 10 to over 1000 kDa [18,19]. The chemical structure of chitosan is similar to that of glycosaminoglycan, the main component of the extracellular matrix (ECM) [20]. Deacetylation degree (DD), molecular mass, solubility, viscosity, crystallinity, flexibility, porosity, tensile strength, and conductivity are frequently evaluated physicochemical properties of chitosan [21,22]. Among them, DD and molecular mass are two of the most important physical characteristics that affect both the chemical and biological properties of chitosan [23,24,25]. In recent years, chitosan preparations with various DDs, molecular masses, and molecular derivatization patterns have attracted much attention because of their potentially beneficial biological properties. Chitosan has various outstanding biological properties, including a good polyelectrolyte action, biodegradability, biocompatibility, bioactivity, antimicrobial property, anticancer property, antioxidant property, cell adhesion properties, non-toxicity, and high flexibility for chemical functionalization [26,27,28]. However, as chitosan is only highly soluble in most diluted acidic solutions at a pH below 6.5 and has a poor solubility in water or most organic solvents, its application field is severely limited [15]. Therefore, improving the solubility of chitosan is a crucial step in extending its scope of application. Deacetylation, chemical modification by adding hydrophilic biomolecules to amino or hydroxyl groups (acylation, carboxylation, alkylation, quaternization, sulfonation, and phosphorylation), crosslinking and chemical or enzymatical depolymerization or degradation are available methods for improving the solubility of chitosan and also optimizing its biological properties [29,30]. Because of the diverse properties of chitosan and its derivatives, they have been extensively applied in the medical and pharmaceutical fields, for example, they have been used in drug delivery [31,32], tissue engineering [20,33], wound management [34,35], gene and cancer therapy [36,37,38], antibiofilm drugs [39], etc. 

## 3. Chitosan-Based Biomimetically Mineralized Composite Materials

In this section, the basic concepts and properties of chitosan-based biomimetically mineralized composite materials are briefly introduced, and then different preparation methods of the composite materials that have been introduced in recent years are presented, mainly discussing the preparations of chitosan organic templates and inorganic minerals in composite materials.

### 3.1. Properties of Composite Materials

Due to its poor mechanical properties and reduced bioactivities, pure chitosan is not suitable for repairing hard tissue defects. Therefore, various chitosan-based organic-inorganic composite materials have been developed for hard tissue repair in recent years, aiming at mimicking the mechanical properties and other biological properties of natural hard tissue. Generally, there are currently two common ways for constructing composite materials of chitosan-inorganics. One way is to mix inorganic substances into chitosan by simple mechanical blending, such as ultrasonic dispersion, and then obtaining the final composite material by freeze-drying [40,41,42]. Nevertheless, the composite material prepared in this way is uneven and heterogeneous due to particle aggregation and a lack of intermolecular interaction between the organic matrix and the inorganics [43]. Another way is to prepare the inorganic layer in situ on the surface of the chitosan scaffold, which acts as a template [44,45,46]. These composite materials are uniform and have excellent mechanical and biological properties [44]. Because of the nearly physiological conditions, without using special equipment or extremely high processing temperature, the latter way also belongs to a biomimetic mineralization technology, which simulates the process of biomineralization [47]. Biomimetic mineralization technology uses organic molecules (protein, peptide, collagen, polymer, etc.) as templates to effectively regulate the nucleation, crystal growth phase transformation, orientation, and particle assembly process of inorganic substances by mimicking the natural process of biomineralization, thus synthesizing organic-inorganic hybrid materials, which simulates natural mineralized tissues, such as bone and teeth [48,49,50]. It can fabricate organic-inorganic composites in a bottom-up way to develop various advanced materials with defined and controlled morphologies and superior mechanical properties under ambient conditions. 

Combined with biomimetic mineralization technology, numerous chitosan-based organic-inorganic biomimetically mineralized composite materials have been prepared using chitosan as an organic template for mineralization, and these composite materials have become a research hotspot in the field of bone tissue engineering and dental hard tissue repair in recent years. In addition to their various excellent biological properties, such as biocompatibility, bioactivity, suitable mechanical strength, slow degradation rate, osteoconductivity, osteoinductivity, etc., a more important advantage of these materials is that the bionic in the structure and function of the organic and inorganic components can be realized to a certain extent. In composite materials, chitosan has a similar chemical structure and biological behaviors to the components of the extracellular matrix in the biomineralization process of teeth or bone, and the inorganic minerals of the composites are usually biogenic minerals, including HAP, other forms of calcium phosphate, such as amorphous calcium phosphate (ACP), dicalcium phosphate dihydrate (DCPD), octacalcium phosphate (OCP), and dicalcium phosphate anhydrous (DCPA), and silica. While pure chitosan scaffolds show some limitations, such as rapid degradation, an insufficient mechanical strength, and a lack of bioactive cell signaling molecules [51], the addition of biominerals, especially HAP, can significantly improve the composite density, fracture toughness, and bioactivity [42]. Furthermore, the minerals prepared by biomimetic mineralization methods highly mimic the morphology, size, and crystallinity of the apatite found in natural bone or teeth, thus achieving composite materials that have a more ideal mechanical and biological activity. Therefore, these chitosan-based biomimetic mineralization composite materials are promising materials for biomedical applications and are starting to attract more of the attention of researchers.

### 3.2. Techniques for the Preparations of Chitosan Organic Templates with Composite Materials

Acting as a template, chitosan can be processed into different forms, like solutions, hydrogels, nanofibers, porous scaffolds, microspheres, nanoparticles, and membranes [52]. In addition to simply preparing chitosan aqueous solutions or using chitosan as a chemical modifier on the surface of other substances, there are currently four other common techniques for preparing chitosan templates, including electrospinning, freeze drying, gelation by physical or chemical crosslinking, and layer-by-layer self-assembly, which are introduced in detail in this section.

#### 3.2.1. Electrospinning

Electrospinning is a simple and efficient technique for fabricating continuous polymer fibers mostly at the micro-scale (>1 μm) or nano-scale (<1000 nm) [53]. The nanofibers created using this method have a large surface area-to-volume ratio, high porosity, and small pore size, which mimic the characteristics of the extracellular matrix [54]. The major components of electrospinning methods are a high voltage power supply, a container (typically a syringe) of polymer solution, a pump, and a collector. A high voltage is applied to a syringe tip or metallic capillary, which is connected to the solution reservoir and pump. Upon the application of a sufficiently high electric field, electrostatic forces overcome the surface tension of the solution to form a jet. Finally, the polymer jet solidifies in nanofibers upon hitting the collector surface [55]. Many researchers have applied this method for the preparation of a chitosan-based scaffold for tissue engineering [56,57,58,59]. For instance, Doan et al. [56] prepared CS nanofibers with uniform diameters using electrospinning and then mineralized the nanofiber surfaces by a wet chemical process. The chitosan/hydroxyapatite (CS/HAP) composite nanofibers promoted osteogenic differentiation by inducing ossification and enhanced the expressions of collagen type I, alkaline phosphatase, osteocalcin, bone sialoprotein, and osterix, showing a considerable potential for use in future bone tissue engineering applications. However, when preparing chitosan organic templates for dental hard tissues, electrospinning technology is rarely used.

#### 3.2.2. Freeze Drying

Freeze drying is one of the most commonly used methods for the preparation of three-dimensional porous polymer scaffolds [60,61,62]. It can transform a solution into solid materials with a sufficient stability for storage and distribution, while retaining the original structure and characteristics of the materials to the maximum extent. The technique involves three steps: freezing of the solution at a low temperature (around −70 °C to −80 °C); a vacuum treatment, which enables the vaporization of the frozen solvent, without passing through the liquid phase, known as sublimation; and applying heat to the frozen product to accelerate sublimation [52]. Many studies have used this technique to obtain porous chitosan-based composite scaffolds for bone tissue engineering [41,63,64]. And a study gained the scaffold of carboxymethyl chitosan (CMC)/ACP nanocomplexes by lyophilizing CMC/ACP gel and used it to re-mineralize dentine in an in vitro tooth model of deep caries [65].

#### 3.2.3. Gelation by Physical or Chemical Crosslinking

Gelation by physical or chemical crosslinking is a common way to prepare chitosan hydrogels. There are two mechanisms of this technique: physically crosslinked hydrogels are formed by intermolecular interaction (electrostatic interaction, hydrogen bonding interaction, and hydrophobic interaction), with small anionic molecules, polyanions, or hydrophobic polymers; and chemical crosslinked hydrogels are formed by the covalent linking of chitosan and other polymers or small cross-linkers through the reaction of their functional groups, where the bond formation is irreversible [66,67]. Belonging to physical crosslinking, ionotropic gelation is one of the most widespread techniques for the preparation of chitosan nanoparticles, which is based on the electrostatic interaction between the amine group of chitosan and a negatively charged group of polyanions, such as tripolyphosphate [68,69,70]. In this method, chitosan is firstly dissolved in acetic acid, then polyanion is added, and nanoparticles are formed spontaneously under mechanical stirring at room temperature [71]. This method has many advantages, such as the simple preparation process, mild preparation conditions, absence of organic solvents, high compatibility, and so on. Chemical crosslinking is often used for the fabrication of chitosan hydrogels with a good mechanical strength and uniform properties, while its preparation process is more complicated, and some crosslinkers have a high toxicity [67]. Poly(lactic acid) (PLA) [72] and poly(ethylene glycol) (PEG) [73] are two biocompatible synthetic polymers that have been used as chemical crosslinking agents to prepare chitosan-based hydrogels. Chitosan hydrogels formed by gelation can provide homogenous 3D scaffolds with good biocompatibility for cell growth and is promising for bone tissue engineering applications [72]. Besides, hydrogel is also a common application form of chitosan used to repair dental hard tissues [74,75].

#### 3.2.4. Layer-by-Layer (LBL) Self-Assembly

Layer-by-layer (LBL) assembly is a highly versatile and simple multilayer self-assembly technique, which can be used to fabricate multilayer coatings with controlled structures and compositions in a variety of biomedical applications, particularly tissue engineering [76,77,78]. In general, the LBL assembly process involves the sequential adsorption of complementary molecules on the substrate surface driven by a variety of interactions involving electrostatic interaction, hydrogen bonding, hydrophobic interaction, and van der Waals interaction [79]. Between the deposition of each layer, washing and drying steps are usually applied to avoid contamination of the next solution due to the adhesion of the previous solution to the substrate and to elute the loose molecules and stabilize them in the formed layer. The desired number of deposited layers can be obtained by repeating the above steps. Besides, many factors like the concentration, ionic strength, and pH can affect the final composition, thickness, and topography of the materials [78]. Liang et al. [80] modified the surface of the electrospun cellulose acetate nanofibers with positively-charged chitosan (CS) and negatively-charged phosvitin (PV) using the LBL self-assembly technique, and then in vitro biomimetic mineralization was carried out through the incubation of the fibrous mats in a simulated body fluid (SBF) solution. The composite scaffold exhibited an excellent cytocompatibility, as well as a good cell adhesion and spreading performance, which make it a promising versatile scaffold for bone tissue engineering. However, LBL assembly technique is less common in dental hard tissue repair than in tissue engineering. 

### 3.3. Biomimetic Mineralization Materials and Techniques for the Fabrication of Chitosan-Based Composites

Inspired by the biomineralization process and biomineral components of natural organisms, the inorganic components that form the biomimetically mineralized composite material with chitosan are usually natural biominerals, and the most common are various calcium phosphates (CaP), such as HAP [81,82], ACP [65,83], DCPD [44], OCP [47], and other intermediate phases of calcium phosphate [84]. Silicas [85,86] are also included. HAP is the major inorganic constituent of natural bone and teeth, with an excellent mechanical strength, biocompatibility, and bioactivity. ACP, DCPD, and other metastable crystalline phases of calcium phosphate are precursors or intermediates during biomineralization, which can finally be transformed into HAP [87]. Silicas are also minerals produced by the biomineralization processes of some organisms. While directly mixing minerals with chitosan in certain proportions can cause 3D scaffold structures, with compositions similar to those of natural bone tissues by phase separation or freeze drying, it is difficult to simulate their microstructure and microenvironment via this method. Therefore, more and more researchers have been concentrating on the biomimetic mineralization technique to fabricate chitosan-based composites. 

**Figure 1:**
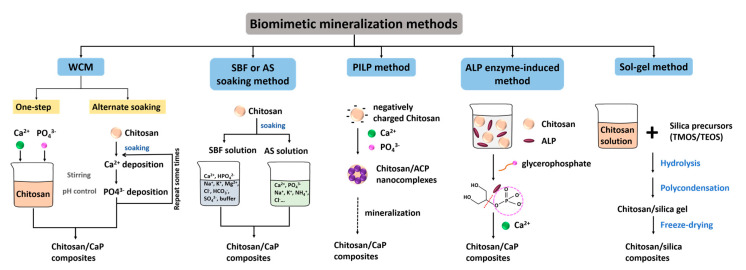
Biomimetic mineralization methods for the fabrication of chitosan-based mineralized composites.

There are five main mineralization methods that use chitosan as an organic template to regulate the nucleation and growth of inorganic minerals, including the wet chemical method, simulated body fluid or artificial saliva soaking method, polymer-induced liquid precursor method, alkaline phosphatase enzyme-induced method, and solution-gelatin method (Figure 1).

#### 3.3.1. Wet Chemical Method (WCM)

WCM is one of the most commonly used methods. It has many advantages, such as simple experimental operations, short preparation time, low working temperature, high purity of products, and low cost [88,89]. WCM precipitation is based on the reaction of soluble calcium salt (Ca(NO_3_)_2_, CaCl_2_, etc.) and soluble phosphates (K_2_HPO_4_, Na_2_HPO_4_, (NH_4_)_2_HPO_4_, etc.). There are two ways to prepare WCM. The first is the one-step process, which involves the simultaneous deposition of calcium and phosphorus. A calcium solution and phosphorus solution of a certain concentration are slowly added to the pre-dissolved chitosan solution simultaneously and incubated for a period of time. In some cases, stirring or pH adjustment is also used in this process [44,72,90]. Another is the alternate soaking process. Briefly, chitosan templates are firstly immersed in a calcium aqueous solution for some time to allow for the deposition of calcium ions and then soaked in deionized water to remove excess ions. Then, they are soaked in a phosphorus aqueous solution to allow for the deposition of the phosphate ion and washed in deionized water. The pH of the reaction system can be controlled using alkaline solutions, and the cycle is repeated a certain number of times [56,73,91,92]. The thickness of the mineral layer on the chitosan templates could be affected by various factors, such as the calcium to phosphorus ratio in the reaction system, soaking time, the cycle repetition times, pH, etc. Therefore, it is speculated that more ideal composites can be obtained by appropriately changing these conditions.

#### 3.3.2. Simulated Body Fluid (SBF) or Artificial Saliva (AS) Soaking Method

The SBF or AS soaking method is also a common way to achieve calcium phosphate precipitation on chitosan templates, which involves soaking chitosan into an SBF or AS solution for a certain amount of time. Conventional SBF is a solution containing 2.5 mM Ca^2+^, 1 mM HPO_4_^2−^ and other components (Na^+^, K^+^, Mg^2+^, Cl^−^, HCO_3_^−^, SO_4_^2−^ and buffer), with ionic concentrations and a pH value similar to those of human blood plasma [93]. In addition to providing a uniform distribution of HAP, a good biocompatibility and bioactivity have been obtained using HA deposition by SBF immersion [94]. However, the fact that it is time-consuming is a disadvantage of the SBF method. It generally takes 7–21 days [80,95]. Therefore, to shorten the immersion time, SBF solutions with different ion concentrations and various times were used, such as the 1.5 times SBF (a 1.5 times ionic concentration of SBF) [80], Modified SBF (a two-fold increase in the concentrations of calcium and phosphate ions, compared to SBF) [47], and 5 times SBF (a 5 times ionic concentration of SBF) [59,95]. AS is an artificial solution that has a similar composition to that of human saliva, its recipes are varied but Ca^2+^ and PO_4_^3−^ are always contained [96]. AS is essential for tooth remineralization, because it supplies calcium and phosphate ions to precipitate HAP. When chitosan-calcium phosphate composites were applied in repairing dental hard tissue, the AS soaking method was usually used [74,75].

#### 3.3.3. Polymer-Induced Liquid Precursor (PILP) Method

The PILP method based on nonclassical crystallization theory is usually applied for the mineralization of bone or dentin collagen scaffolds, and chitosan acts as a polymer template for stabilizing ACP precursors [65,83]. The ACP precursors stabilized by chitosan could be transferred into collagen fibers and finally transformed into HAP nanocrystals, thus achieving the intrafibrillar mineralization of collagen. The key of the PILP process is to find negatively charged polymers for stabilizing amorphous precursor particles in order to achieve a liquid-liquid phase separation. It has been demonstrated that carboxymethyl chitosan (CMC), the derivative of chitosan enriched in carboxyl groups, could stabilize ACP to form liquid-phase nanocomplexes of CMC/ACP, which could aid in the intrafibrillar mineralization of collagen and thereby facilitate the remineralization of demineralized dentine [83].

#### 3.3.4. Alkaline Phosphatase (ALP) Enzyme-Induced Method

Alkaline phosphatase (ALP) is highly expressed in the cells of mineralized tissue and plays a vital function in bone mineralization. It releases inorganic phosphate from the mineralization inhibitor inorganic pyrophosphate, thus promoting extracellular mineralization [97]. Inspired by this function of ALP, the ALP enzyme-induced mineralization method was applied in the mineralization of chitosan scaffolds. The method involves the ALP that already existed in chitosan gels or scaffolds providing phosphate ions by cleaving the organic glycerophosphate and then phosphate ions reacting with the Ca^2+^ in the system to form calcium phosphate precipitates, thereby constructing chitosan organic-inorganic mineralized composites [64,98]. It was reported that the slow mineralization induced by the ALP enzyme could form a much denser and more uniform nano HAP deposition in chitosan scaffolds, and the mineralized scaffolds promoted the osteogenic differentiation of pre-osteoblasts in vitro and demonstrated an excellent tissue integration in vivo [64].

#### 3.3.5. Solution-Gelatin (sol-gel) Method

Silicon and silica are fundamental inorganic components that are widely found in organisms. Organisms produce silica by silica biomineralization (also called biosilicification) through biological macromolecule-mediated self-assembly. This self-assembly process is a “bottom-up” construction of nanostructured hierarchical silica materials with complex three-dimensional architectures [99]. Inspired by this, the biomimetic synthesis of silica-relevant hybrid materials using chitosan as a template via the silica biomineralization processes is attracting an increasing amount of attention, and the solution-gelatin (sol-gel) technique is a universal mineralization technique [85,86,100]. The sol-gel method is indeed a wet-chemical technique with two reactions: hydrolysis and polycondensation [101]. In general, polymeric molecules and metal-organic alkoxides, especially tetramethoxysilane (TMOS) and tetraethoxysilane (TEOS), are sol precursors to silicates, and the final gel of an integrated three-dimensional network structure can be obtained by sol-gel transition [102]. It was shown that silicon–chitosan hybrid hydrogels could be obtained by the biomimetic sol-gel mineralization of chitosan under mild conditions, with no catalyst or any organic solvent [100]. Besides, studies demonstrated that silicon–chitosan hybrid scaffolds can also act as a versatile template for the formation of apatite and may therefore be promising candidates for bone tissue engineering applications [85,86].

## 4. Applications of Chitosan-Based Biomimetically Mineralized Composite Materials in Human Hard Tissue Repair

Because of the excellent biological properties of various chitosan-based biomimetically mineralized composite materials, they have been extensively applied in the fields of human hard tissue repair, including bone tissue engineering and dental hard tissue repair.

### 4.1. Applications in Bone Tissue Engineering

Natural bone exhibits a hierarchical structure, mainly consisting of multilayered collagen fibers and the inorganic component, HAP [103]. In consideration of events such as trauma, infection, and tumor, which cause the large-scale destruction or loss of bone tissue, exploring materials that can replace or even reconstruct bone structure is an urgent challenge in orthopedic clinical practice. Bone tissue engineering is currently a hot research field and aims to realize bone reconstruction and regeneration, focusing on scaffolds, cells, growth factors, and their interrelation in a microenvironment [104]. For a bone tissue engineering scaffold to be successful, it must be highly porous, osteoconductive, biodegradable, biocompatible, mechanically strong, and capable of efficiently guiding new bone formation in the defect [105]. Preparing organic-inorganic composite nanofibers to simulate the composition of the ECM is an effective strategy for providing bone tissue engineering scaffolds. According to the structure and composition of natural bone, natural macromolecule/HAP composite scaffolds synthesized by biomimetic mineralization with natural bioactive macromolecules are currently key research focuses in this field [59,106,107]. Among these macromolecules, chitosan is a popular alternative because of its excellent biological properties [108]. 

Different chitosan-based organic-inorganic composite materials using the biomimetic mineralization technique and their important properties in the field of bone tissue engineering are presented in Table 1. Using a wet chemical method, Doan et al. prepared chitosan/hydroxyapatite (CS/HAP) nanofibers with a homogeneous HAP deposit [56]. The composite nanofibrous scaffold promoted osteogenic differentiation by inducing ossification and enhanced the expressions of collagen type I, alkaline phosphatase, osteocalcin, bone sialoprotein, and osterix, thus showing that it has considerable potential in bone tissue engineering applications. Compared with ordinary chitosan, carboxymethyl chitosan (CMCS) has a better water-solubility, biodegradability, and bioactivity, which allows CMCS to chelate Ca^2+^ and induce the deposition of apatite [109,110]. HAP-coated electro-spun CMCS nanofibers prepared by biomimetic mineralization using 5 times simulated body fluid increased the ALP activity and the gene expression level of Runx2 and ALP and promoted new bone formation and maturation [59]. In order to fabricate a hybrid nanostructured HAP-CS composite scaffold with HAP nanorods perpendicularly-oriented to CS fibers, Guo and his co-workers [44] applied a two-stage preparation process using brushite (DCPD, CaHPO_4_·2H_2_O) as transitory precursors and mimicked the biomineralization process of the apatite in bone tissue. The process included the deposition of DCPD on the CS fiber porous scaffold using a dip-coating method and the formation of a hybrid nanostructured HAP-CS composite scaffold through the in-situ conversion of DCPD into HAP using a bioinspired mineralization process. The composite scaffold exhibited good mechanical properties and could support the adhesion and proliferation of hBMSCs. Moreover, it could promote the formation of new bone in rat calvarial defects. To further improve the mechanical and biological properties of chitosan-HAP composite scaffolds, graphene was also introduced into the composite scaffolds. Graphene and its derivatives, such as graphene oxide (GO) and reduced graphene oxide (RGO), are highly biocompatible and can easily be functionalized by various organic and inorganic compounds due to the presence of various functional side groups (hydroxyl, carboxyl, and epoxides) on its surface [111,112]. It was found that extensive mineralization occurred in the CS-GO conjugate system because of strong electrostatic interactions between the functional groups (carboxyl groups of GO and amino groups) of CS and calcium ions in an SBF solution. The combination of a chitosan–graphene oxide conjugate and biomimetic mineralization was advantageous in favorably modulating cellular activity. It induced homogeneous spatial osteoblastic cell growth and increased mineralization [95]. Another study showed that chitosan acted as an interfacial soft polymeric template on the surface of RGO, promoting an ordered growth of the hydroxyapatite particles. The three-component composite mineralized scaffold mimicked the structure and composition of natural bone and exhibited a relatively higher rate of cell proliferation, osteogenic differentiation, and osteoid matrix formation [113]. 

In addition to the abovementioned composite materials, which contain chitosan as the only organic template, in recent years, researchers have also combined chitosan with other polymers to prepare multi-component biomimetically mineralized scaffold materials. In the field of bone tissue engineering, collagen (Col) is usually a good natural polymer for forming a hybrid scaffold with chitosan. Wang et al. used CMC as a polyelectrolyte template to stabilize ACP in order to form nanocomplexes of CMC/ACP and then fabricated mineralized collagen scaffolds using a biomimetic method based on the polymer-induced liquid precursor process. They found that nanocomplexes of CMC/ACP significantly increased the modulus of the collagen scaffolds, and the scaffolds could better promote the regeneration of bone tissue in defects [83]. Another similar study also prepared CMC/ACP nanocomplexes under acidic conditions (pH < 3.5) and realized biomimetic synchronous self-assembly/mineralization (SSM) of a collagen scaffold [114]. Zou et al. compounded three matrix materials (CS, Col, and PLA) uniformly, with the assistance of sonication and amidation to regulate the in-situ crystallization of nHAP in order to fabricate CS/Col/PLA/nHAP scaffolds. The scaffolds improved the mechanical properties and the formation of crystals in the SBF, and it had a good biocompatibility and could maintain the cell growth [72]. Moreover, gelatin, a protein derived from collagen with a similar structure to collagen, is a biodegradable biopolymer with a high biocompatibility [115], which has also been widely used in biomimetic composite scaffolds with chitosan [91,116,117]. A gelatin-chitosan core-shell structured nanofibers mat with a three-dimensional porous structure was fabricated by a coaxial electrospinning technique. An arginine-glycine-aspartic acid (RGD)-like structure was formed to mimic the organic component of the natural bone extracellular matrix, and then homogeneous HAP was deposited on its surface using a wet chemical method. The biomimetic composite scaffolds could further enhance osteoblast cell proliferation [91]. Heinemann et al. prepared organically modified hydroxyapatite (ormoHAP) in gelatin gels using the double migration technique and mineralized chitosan porous scaffolds created using the Net-Shape-Nonwoven (NSN) technique. The mineralized NSN-scaffolds improved the attachment, proliferation, and differentiation of hBMSC, presenting a remarkable application potential for bone tissue engineering [116]. In addition to collagen and gelatin, other biodegradable polymers or proteins, such as cellulose [80], PLA [118], PEG [73], and silk fibroin [92], were also effective organic additives, acting as crosslinking agents or scaffolds of the chitosan-based composite materials. While various chitosan/calcium phosphates are the most common biomimetically mineralized composite materials used in bone tissue engineering, some chitosan/silica biomimetically mineralized scaffolds have also been applied due to their capability in inducing the formation of apatite and good potential for promoting new bone regeneration [85,86].

### 4.2. Applications in Dental Hard Tissue Repair

Dental caries, acid erosion, and trauma are common causes of the demineralization and defects in teeth. Conventional treatments, such as remineralization treatment using fluoride agents and restorations with materials of metals, composite resins, or ceramics, cannot restore the original structure and properties of teeth. Therefore, exploring new biomimetic materials that can promote the remineralization of dental hard tissues and reconstruct the structure of enamel or dentin is a major research direction in relation to the repairing of dental hard tissues [12,119]. In recent years, chitosan has been used as a carrier or stabilizing agent of calcium phosphates due to its excellent biological properties and further combined with biomimetic mineralization technology. The composite materials obtained in this way are widely applied in dental hard tissue restorations, especially in the biomimetic remineralization of enamel and dentin. 

Recent studies on the application of chitosan-based composite materials combined with the biomimetic mineralization technique in repairing dental hard tissue are presented in Table 2. Xiao et al. synthesized CMC/ACP nanocomplexes using CMC as a stabilizer of ACP, and with the help of NaClO and chimaeric peptide, which mimics amelogenin, well-organized enamel-like crystals, equipped with strong mechanical properties, formed on the demineralized enamel surface [120]. A biomimetic CS-HAP hybrid coating was fabricated under CS-Emdogain hydrogel action by in situ biomimetic mineralization. The coating that was mainly composed of carbonate-substituted HAP, B-type, with a c-axis orientation, and presented a highly organized enamel-like structure may be a promising hybrid material for enamel remineralization [75]. Furthermore, Simeonov et al. developed novel hybrid chitosan/calcium phosphate microgels using chitosan microgels as a template for calcium phosphate in situ deposition. The hybrid materials have several advantages as a re-mineralizing agent, including bio-adhesiveness, antimicrobial properties, and a continuous supply of calcium and phosphate ions to ensure the successful remineralization of the model’s initial caries lesions [121].

Chitosan was also introduced into agarose hydrogel to induce a continuous compact CS-HAP composite layer growing on the etched dentinal surface, showing a beneficial effect in the remineralization of etched dentine [74]. Since the intrafibrillar mineralization of collagen is an important goal of dentin remineralization [122], CMC was used as the analog of acid non-collagenous protein and acted as the template for stabilizing ACP to form CMC/ACP nanocomplexes, thus further accomplishing the intrafibrillar mineralization of collagen. Besides, the completely demineralized dentine was partially re-mineralized in an in vitro tooth model of deep caries, suggesting that CMC/ACP nanocomplexes may be a potential indirect pulp capping material for the management of deep caries during vital pulp therapy [65]. Another study modified collagen fibers with phosphorylated chitosan and created new nucleation sites for ACP on the fiber, thus promoting the intrafibrillar mineralization of collagen through the PILP mineralization process and achieving the remineralization of calcium-depleted dentin within 96 h [123]. 

## 5. Conclusion

Nowadays, various synthetic composite materials are becoming more popular than human-derived biomaterials in the field of tissue engineering. Although biomaterials, such as decellularized ECM, have similar components and structure to natural ECM of organisms, they have some limits such as complicated preparation technique, possible immunogenicity and mechanical properties that are difficult to control [124]. In contrast, the structure and mechanical properties of synthetic composite materials can be manipulated and controlled, and the presence of some components in synthetic materials can endow them with specific biological properties [125].

As a natural cationic biopolymer with a unique molecular structure and various excellent biological properties, chitosan is a suitable organic template or scaffold for regulating mineral formation during the biomimetic mineralization process. It can thereby form biomimetically mineralized composite materials with various advantages (good bionic characteristics, mechanical properties, biocompatibility, bioactivity, and osteogenic potential), and these materials have been widely applied in the fields of bone tissue engineering and dental hard tissue repair. In different studies, different results were found for these chitosan-based biomimetically mineralized composite materials regarding, e.g., the preparation techniques of chitosan organic templates, methods of biomimetic mineralization, types of inorganic minerals, and application forms of composites. These are also the key factors affecting the final properties and functions of the composite. By changing or optimizing these factors, it may be possible to prepare composite materials with better biocompatibility, bioactivity, osteoinductivity, mechanical properties and bionic abilities, which may be promising products for clinical applications. In recent years, in order to optimize the performance of chitosan-based biomimetically mineralized composite materials, simple two-component composite materials have been investigated by researchers but have been unable to meet their expectations. Thus, many studies have incorporated other organic or inorganic components into composite materials and prepared multi-component biomimetically mineralized composite materials with multiple biological functions. This may be a major development direction for the development of chitosan-related biomimetically mineralized composite materials in the future.

## Figures and Tables

**Table 1:** Applications of chitosan-based biomimetically mineralized composite materials in bone tissue engineering.

Chitosan or Its Derivatives	Composite Forms	Minerals	Other Organic/Inorganic Components	Preparation Techniques of Chitosan Template	Methods of Biomimetic Mineralization	Important Properties	Reference
Chitosan	Nanofibers	HAP	-	Electrospinning	Alternate soaking of WCM	Promoted osteogenic differentiation by inducing ossification	[56]
Carboxymethyl chitosan	Nanofibers	HAP	-	Electrospinning	Soaking in 5 times SBF solution	Increased the ALP activity, promoted the gene expression level of Runx2 and ALP, promoted new bone formation and maturation	[59]
Chitosan	Porous scaffolds	DCPD, HAP	-	Needle-punching process	dip-coating method and in situ precipitation by WCM	Excellent biocompatibility, osteoinductivity and mechanical properties	[44]
Chitosan	Membranes	HAP	GO	Chemical conjugation with GO	Soaking in 5 times SBF solution	Influenced osteoblastic cell differentiation, mineralization, and cell growth	[95]
Chitosan	Aerogel networks	HAP	RGO	Functionalize RGO	Soaking in 1.5 times SBF solution	Exhibited relatively higher rate of cell proliferation, osteogenic differentiation and osteoid matrix formation	[113]
Carboxymethyl chitosan	Nanocomplexes	ACP	Collagen	Dissolved in water	PILP method	Promoted the proliferation and differentiation of mouse preosteoblasts, accelerated the regeneration of bone in the defects of rat calvaria bone	[83]
Chitosan	Porous scaffolds	nHAP	Collagen, PLA	Emulsion-crosslinking	WCM	Improved the mechanical properties and the formation of crystals in SBF, had good biocompatibility, maintained the cell growth	[72]
Chitosan	Core-shell structured nanofibers	HAP	Gelatin	Coaxial electrospinning technique	WCM	Enhanced osteoblast cell proliferation	[91]
Chitosan	Fibers	HAP	Gelatin	Net-Shape-Nonwoven (NSN) technique	Double migration technique	Improved attachment, proliferation, and differentiation of hBMSC	[116]
Chitosan	Nanofibers	HAP	Cellulose, phosvitin	LBL self-assembly technique	Soaking in 1.5 times SBF solution	excellent cytocompatibility, as well as good performance of cell adhesion and spreading	[80]
Chitosan	Fibers	HAP	PLA	Modification on electrospun PLA nanofiber	Soaking in 10 times SBF solution	Mimicked structural, compositional, and biological functions of native bone	[118]
Chitosan	Hydrogel	HAP, DCPD	PEG	Chemical crosslinking with PEG	Alternate soaking of WCM	Induced excellent cell adhesion ability	[73]
Chitosan	Porous scaffolds	HAP	Silk fibroin	Freeze drying	Alternate soaking of WCM	Good mechanical property, promoted early cell attachment and enhanced osteogenic differentiation	[92]
Chitosan	Porous scaffolds	nHAP	ALP	Freeze drying	ALP enzyme-induced mineralization method	promoted the osteogenic differentiation of pre-osteoblasts in vitro and demonstrated excellent tissue integration in vivo	[64]
Chitosan	Thermosensitive hydrogels	CaP	ALP	Gelation	ALP enzyme-induced mineralization method	Promoted mineralization, may be suitable materials for bone replacement.	[98]
Chitosan	Hybrid scaffolds	Silica	-	Freeze drying	Sol-gel process	No cytotoxicity, excellent in vitro bone bioactivity	[85]
*N*-guanidinium-chitosan acetate	Hybrid scaffolds	Silica	-	Freeze drying	Sol-gel process	Acted as versatile templates for biomineralization, inducing the formation of HAP	[86]

Hydroxyapatite (HAP); wet chemical method (WCM); simulated body fluid (SBF); alkaline phosphatase (ALP); dicalcium phosphate dihydrate (DCPD); graphene oxide (GO); reduced graphene oxide (RGO); amorphous calcium phosphate (ACP); polymer-induced liquid precursor (PILP); nanohydroxyapatite (HAP); layer-by-layer (LBL); poly(lactic acid) (PLA); poly(ethylene glycol) (PEG), calcium phosphate (CaP).

**Table 2:** Applications of chitosan-based biomimetically mineralized composite materials in dental hard tissue repair.

Chitosan or Its Derivatives	Composite Forms	Minerals	Other Organic/Inorganic Components	Preparation Techniques of Chitosan Template	Methods of Biomimetic Mineralization	Important Properties	Reference
Carboxymethyl chitosan	Nano-complexes	ACP	Chimaeric peptides	Dissolved in water	PILP method, immersing in AS solution	Promoted rapid biomimetic remineralization of the demineralized enamel, formed well-organized enamel-like crystals	[120]
Chitosan	Coating	Carbonate-substituted B-type HAP	Emdogain	Gelation	Immersing in AS solution	Provided highly organized enamel-like structure for teeth remineralization	[75]
Chitosan	Hybrid microgels	Amorphous CaP and poorly crystalline carbonated B- type HAP	-	Ionotropic gelation	in situ precipitation by WCM	Owned several advantages as remineralizing agent, including bio-adhesiveness, antimicrobial properties as well as continuous supply of calcium and phosphate ions to ensure the successful remineralization of the model initial caries lesions	[121]
Chitosan	Coating	B-type Ca-deficient HAP	Agarose	Gelation	Immersing in AS solution	Showed a benefic effect on remineralization of etched dentine	[74]
Carboxymethyl chitosan	Nanocomplexes	ACP	Collagen	Dissolved in water	PILP method, immersing in SBF solution	Partially remineralized the completely demineralized dentine in an in vitro tooth model of deep caries, a potential indirect pulp capping material	[65]
Phosphorylated chitosan	Fibers	ACP	Collagen	Chemical modification on collagen fibers	PILP method	Promoted intrafibrillar mineralization of collagen, achieved remineralization of calcium-depleted dentin within 96 h	[123]

Amorphous calcium phosphate (ACP); polymer-induced liquid precursor (PILP); artificial saliva (AS); calcium phosphate (CaP); wet chemical method (WCM); simulated body fluid (SBF).
